# Perceptions on the importance of gerontological education by teachers and students of undergraduate health sciences

**DOI:** 10.1186/1472-6920-7-1

**Published:** 2007-01-19

**Authors:** Víctor Manuel Mendoza-Núñez, María de la Luz Martínez-Maldonado, Elsa Correa-Muñoz

**Affiliations:** 1 Universidad Nacional Autónoma de México, Unidad de Investigación en Gerontología (FES ZARAGOZA). Batalla 5 de mayo s/n, esq. Fuerte de Loreto, Col. Ejército de Oriente, 09230 México, D. F., México

## Abstract

**Background:**

The main challenge of higher education institutions throughout the world is to develop professionals capable of understanding and responding to the current social priorities of our countries. Given the utmost importance of addressing the complex needs of an increasingly elderly population in Mexico, the National Autonomous University of Mexico has systematically incorporated modules dealing with primary gerontological health care into several of its undergraduate programs in health sciences. The objective of this study was to analyze teacher's and student's perceptions about the current educational practices on gerontology.

**Methods:**

A cross-sectional study was carried out with a sample of 26 teachers and 122 undergraduate students. Subjects were administered interviews and responded survey instrument.

**Results:**

A vast proportion of the teachers (42%) reported students' attitudes towards their academic training as the most important factor affecting learning in the field of gerontology, whereas students reported that the main problems of education in gerontology were theoretical (32%) and methodological (28%). In addition, 41% of students considered education on ageing matters as an essential element for their professional development, as compared to 19% of teachers (p < 0.05).

**Conclusion:**

Our findings suggest that the teachers' perceptions about the low importance of education on ageing matters for the professional practice of health sciences could be a negative factor for gerontology teaching.

## Background

The demographic and epidemiological transitions throughout the world make it necessary to train professionals in the area of health sciences with a biological, psychological and social perspective about the ageing process [1-4]. Several studies in the field have emphasized the decisive role that students' perceptions and attitudes about ageing play in the achievement of educational outcomes [5-8]. However, the role of the teacher in this process has not been analyzed.

The teaching-learning process is a lived experience involving the engagement with others in the acquisition of knowledge. It also includes the multidimensional processes of expanding one's imagination, naming the new, keeping up-to-date with advancements and shifts in the field, abiding paradox, constructing meaning, and getting involved in a continuous dialogue. In this sense, learning cannot be free from the context. Learning and its organization into an individual's personal knowledge system are highly dependent on the context in which things have been learned. Likewise, the intrinsic motivations associated with students' and teachers' perceptions are associated to their success in the process [9,10].

Perception is understood as the positions, attitudes, affects and behaviors that an individual exhibits towards certain processes, situations, objects or persons. Current research has shown that the myths, stereotypes and prejudices towards ageing held by some undergraduate students have a negative repercussion on their academic education, and in turn, over the quality of services and attention that they give to elderly populations [11-14].

The paramount importance of promoting a deep understanding of the ageing process and of the medical, social and psychological needs of the elderly population, is now widely acknowledge in our country. Hence, the National Autonomous University of Mexico at Zaragoza has incorporated into the current undergraduate health sciences education program theoretical content in the field of gerontology. However, there has been a lack of systematic training for teachers in these areas (only 1% of the teachers in charge of these courses hold graduate studies in the field).

The purpose of this study was to evaluate the perception of students and teachers towards current educational programs in gerontology. The understanding of such perceptions is expected to become an important element in the development of educational strategies leading to the promotion of higher standards in the provision of health and social services for the elderly. This appreciation would also entail the enforcement of positive attitudes towards ageing and the teaching of the gerontology in our graduates.

## Methods

### Design and subjects

A cross-sectional study was carried out with a sample of 26 teachers and 122 undergraduate students of health sciences, with prior informed consent.

All participants were administered a questionnaire evaluating their previous education in geriatrics and gerontology and their perspectives about educational priorities in gerontology for professional practice.

Issues associated to teaching of gerontology were defined as the significant difficulties for acquisition of theoretical knowledge, technical skills, procedures, personal relationships, and autonomous learning during the teaching-learning process of gerontology. In this regard, the main issues considered in the evaluation of teachers' and students' perceptions which make gerontological education difficult were classified as follows:

(i)Theoretical: significant limitations of scientific knowledge and their own knowledge on gerontology; (ii) Methodological: significant limitations of the definition and establishment of objectives, content and procedures for the attainment of stated objectives for gerontological education; (iii) Motivational: little interest or dissatisfaction with conduction of academic activities linked to gerontology; (iv) Attitudinal: negative behavior and little participation of individuals during training, which significantly affects their professional performance in the gerontological area; (v) Access to Literature: technical and infrastructure limitations which significantly affect accessibility to gerontological bibliography; and (vi) Previous Education: having taken previous formal extracurricular courses on basic gerontological knowledge.

The questionnaire for evaluating their previous education in geriatrics and gerontology and their perspectives about educational priorities in gerontology for professional practice was previously pilot-tested in a group of 10 students so as to verify the understanding of the questions. The definitions of all the study variables were included. The questionnaire was approved by a panel of 4 gerontologists. It was personally and directly given to teachers; and in small groups (10–12 persons), to students during the inter-semester period.

Additionally, in order to deepen our understanding of current perceptions underlying current teaching practices in the field of gerontology, an interview with a semi-structured guide was administered to 10 students -5 from beginning semesters and 5 from advanced semesters- and to 5 teachers -one from each degree course. Interviews lasted 30 minutes on average, and they were recorded.

### Data analysis

The data were analyzed using the SPSS 12.0 statistical program (SPSS Inc, Chicago, IL, USA). Descriptive statistics were means and percentages. Chi-square testing (X^2^) was used to compare proportions.

### Qualitative analysis

Interviews were transcribed and analyzed in accordance to the scheme proposed by Huberman and Miles (1994) [15]: reading, coding, presentation, reduction and interpretation. Information was coded in the following categories regarding the priorities of gerontological education: A) Biological Aspects of Ageing; B) Sociological Aspects of Ageing; C) Psychological Aspects of Ageing; and D) Clinical Practice in the Elderly.

## Results

### Description of the participants

The average age of teachers was 44 ± 6.8 years. 65% were women and 35%, men. In regards to their academic background, 7 were chemist-pharmacist-biologists, 6 psychologists, 6 dentists, 4 surgeons and 3 nurses.

Average age of students was 22 ± 2.7 years. 71% were women and 29%, men. 49 were students registered in the first four semesters (elementary level) of training and 73 from fifth to ninth semester (advanced level) of undergraduate programs of dentistry (69), medicine (33) and nursing (20).

### Problems affecting teaching

A vast proportion of the teachers (42%) reported students' attitudes towards their academic training as the most important factor affecting learning in the field of gerontology, followed by their lack of previous education (36%). Contrary to the above, students' reported that the biggest problem associated with gerontology learning was related to theoretical (32%) and methodological aspects (28%) as shown in Table 1.

**Table 1:** Main problems associated with current teaching practices in gerontology

Problems	Students N (%)	Teachers N (%)
Theoretical	39/122 (32)*	2/26 (8)
Methodological	34/122 (28)*	2/26 (8)
Motivational	18/122 (15)	4/26 (15)
Attitudinal	16/122 (13)	11/26 (42)**
Access to literature	15/122 (12)	0/26 (0)
Previous education	11/122 (9)	9/26 (36)**

Chi-square test: *p < 0.05; **p < 0.01

### Importance of Education in Gerontology

As to the perceived priority areas within gerontology education, an agreement was evident among students and teachers on the importance of biological content, whereas social and research matters were considered the least important (Table 2).

**Table 2:** Disciplines considered as most important in gerontological education

Discipline	Students N (%)	Teachers N (%)
Biology	48/122 (39)	16/26 (61)*
Psychology	30/122 (25)*	1/26 (4)
Bioethics	15/122 (12)	5/26 (19)
Research	15/122 (12)	1/26 (4)
Anthropology	8/122 (7)	0/26 (0)
Sociology	6/122 (5)	3/26 (10)

Chi-square test: *p < 0.05

With regard to the importance of the integration of theoretical content in gerontology within the current academic programs in the fields of biological, health and behavioral sciences, results showed that both students (57%) and teachers (58%) acknowledged the importance of both disciplines for their professional development. However, there was an important difference between the percentages of students (41%) that considered education on ageing matters as an essential element for their professional development, as compared to a lower percentage of teachers (19 %) that considered it as essential (Table 3). In this regard, significant differences were not found by gender or school level (p > 0.05).

**Table 3:** Perceptions related to the importance of gerontology in health sciences

Perception	Students N (%)	Teachers N (%)
It is important to include gerontological content in the curriculum	69/122 (57)	15/26 (58)
Education on ageing matters is essential in my profession	50/122 (41)*	5/26 (19)
Academic content on ageing matters is relevant in the teaching of health sciences	18/122 (15)	2/26 (8)
Teachers need further training on ageing matters	39/122 (32)	11/26 (42)
The direct contact with elderly populations is essential for students in health sciences	79/122 (65)	13/26 (50)
Teachers should pursue postgraduate studies in geriatrics and gerontology	44/122 (36)	12/26 (46)
There is a need for interdisciplinary work in the field of gerontology	55/122 (45)*	6/26 (23)
I do not like to work with elderly populations	4/122(3)	1/26 (4)

Chi-square test: *p < 0.05

It is important to note that the percentages of perceived relevance of ageing related content within the curriculum are higher in the case of students (15%) when compared to teachers (8%). Furthermore, students (45%) were more emphatic than teachers (23%) regarding the importance of direct work with elderly populations as well as the need to promote interdisciplinary approaches in the field (Table 3).

The analysis of teachers' responses made evident two main positions towards ageing. Several teachers (60%) conceptualized ageing as an illness:

" [Ageing] is a final stage in the life of an individual like a disease."

*"We have to concentrate on the clinical attention to this age group [the elderly], because ageing is like another additional disease"*

By the same token, 40% of teachers conceptualized ageing as a gradual and multi-determined process, stressing the importance of interdisciplinary work in this area and highlighting the need to concentrate in the affective area in order to sensitize their students towards the social, biological, cultural and medical aspects of ageing. Examples of the perspectives endorsed by the second group are the following:

*"I try to introduce my students to basic concepts about ageing so that they can overcome myths and taboos about the elderly that influence the way they approach them"; "I try to sensitize my students to the needs of the elderly, to approach them with due respect, to make them understand the anatomical and functional changes associated to ageing, and to develop a holistic perspective towards their treatment"*.

In general, teachers emphasized the importance of relating theory to practice in the education field, stressing the importance of the latter.

### Teachers' priorities in the teaching of gerontology

In order to gain insight into the teachers' perceived priorities in the teaching of gerontology, university teachers were asked the following question: What do you think is the most important area in the teaching of gerontology?. Responses to this question were analyzed so as to qualitatively identify different categories of descriptions of teachers' priorities. Four main priorities emerged from the present study: biological, sociological, and psychological correlates of ageing as well as matters related to the clinical practice with elderly populations. Brief descriptions of those categories and representative excerpts from the teacher survey are summarized in Table 4.

**Table 4:** Categorization of teaching priorities in gerontology

Priority	Representative excerpts	
	Teachers	Students

A. Biological aspects of aging	"Students don't have interest on this issue." "Training in this area is necessary in order to give appropriate training to the students." "Subjects related to biology are essential in geriatrics and gerontology."	"Teachers don't have enough knowledge about this topic." "This is a very difficult topic in the teaching-learning process." "It is very important to deal with this topic." "It is very important to include these topics in training." "Extending topics with information regarding these themes." "Extending these topics to get more knowledge." "It is important to get to know the diseases and complications in the elderly."
B. Sociological aspects of aging	"Many students consider that social gerontology is not relevant to their clinical practice." "Almost all students in health sciences consider the social aspects as a tedious topic" "We need to be prepared for a gerontological culture."	"Any stage of human development is of great importance both at the individual and sociological levels." "The social area is important for elderly care." "We need to know the social impact of this part of society."
C. Psychological aspects of aging	"Some students consider that the elderly are complicated patients and thus avoid them" "Some teachers don't have enough knowledge on the psychology of aging, and therefore the students do not have it either"	"Many elderly persons suffer depression and mild cognitive impairment, and students don't have enough knowledge for their diagnosis and treatment." "It is important to have information about clinical psychology to take care of the elderly." "It is important to know about the behavior of the elderly."
D. Clinical practice in the elderly	"It is important to increase clinical practice with the elderly." "We treat elderly patients without knowledge, thus it is important to train teachers in this area."	"There should be more clinical practice included; more than 70% of patients are elderly." "Faculty teaching these topics should focus more on the clinical area."

The categories were analyzed in terms of the teachers' objectives and their strategies for achieving them. The priorities described in categories A, B and C show an interest in understanding the theoretical elements related to the biology, sociology and psychology of aging while category D shows an intention to promote a relational and coherent understanding of gerontology for clinical practice. Therefore, a qualitative shift in intention is evident from the emphasis in the teaching of theoretical knowledge (categories A, B and C) to the interest in aspects related to the actual clinical practice.

## Discussion

The demographic and epidemiological transition many Latin-American Countries, such as Mexico, are going through, urges Universities to train professionals for elderly care. Thus, the interest on the gerontological field among undergraduate students is to be promoted. In this area, a study conducted in the University of Western Ontario, Canada, found that less than 20% of first-year medical students were interested in geriatrics, and in the second-year the percentage dropped to 16% [16].

In this regard, in our study, students were asked: *Is education on ageing matters essential in your profession? *41% of respondents said yes, whereas only 19% of teachers agreed with such a statement. This suggests that even though a large percentage of the students acknowledge the importance of gerontology in their professional practice, the perception of teachers may negatively influence the students' interest.

Therefore, it is necessary to implement training and refresher courses to strengthen the perception of teachers regarding the importance of gerontology in the professional practice of health sciences, since a large percentage of the faculty has erroneous concepts, and even thinks that *"ageing is a disease"*.

Some researchers refer to the aforementioned attitudinal profile as "ageism" and suggest that it can only be overcome on the basis of intensive teacher education programs [5,8,13]. In connection with this, the teachers' perception in as to the little importance of education on aging matters for the medical profession could be linked to certain degree of *ageism*. However, this would have to be proven in future studies.

Additionally, it has been reported among medical school students that females are more interested in geriatrics than males [16]. This suggests that the interest in specialized health care for the elderly has certain gender influence, perhaps determined by socio-cultural aspects which relate more frequently the care of the elderly with women.

In our study, no significant differences were found by gender in as to the students' perception regarding the importance of gerontology for their professional practice. This response might be due to the fact that a considerable number of patients taken care of by health sciences students during their clinical practice are elderly, thus the latter acknowledge the importance of having training in this area, regardless of the fact that they would or would not like to be geriatricians or gerontologists.

Another relevant finding of this study was the teachers' reports indicating the important role that students' attitudes and lack of previous education have as limiting factors in the development of instructional approaches in this field. Such finding is consistent with previous research studies [6,7,17].

Vis-à-vis this situation, it is indispensable to implement curricula which foster knowledge and ongoing education in the gerontological field. In addition, it is necessary to have interactions with the elderly in their professional practice in order to improve the attitudes and maintain the students' interest in this field through tutorial teaching. In this sense, it has been showed that the isolated experimental training loses its beneficial effect on knowledge, attitudes and interests after a year [18].

As to the emphasis given by teachers and students to the education on biological aspects, above social and research topics and issues, such perspective may be rooted in traditional cultural and educational views. These tend to approach ageing from an eminently biological perspective, and put aside other levels of analysis. Likewise, the very limited relevance ascribed to research can be understood as an evidence of the scarce or even null involvement of teachers in research-related activities, and also of the early stage of development of this field.

## Conclusion

Our findings suggest that the teachers' perceptions about the low importance of education on ageing matters for the professional practice of health sciences could be a negative factor for gerontology teaching in undergraduate students of health sciences of the National Autonomous University of Mexico (at ZARAGOZA Campus).

Also, the little importance given to geriatrics or gerontology training by faculty members might be due, to a large extent, to the lack of academic training of teachers in such areas, since only 1% of them have graduate studies in ageing. For this reason, it is essential that teachers teaching the subjects of geriatrics and gerontology have sound academic training in the study field of ageing.

Additionally, it would also be convenient to implement extracurricular courses in order to improve the perception of the health sciences graduate students on the importance of geriatrics and gerontology training. Also, it is necessary to evaluate the influence of ageism of teachers and students in the geriatrics and gerontology teaching-learning process.

## Competing interests

The author(s) declare that they have no competing interests.

## Authors' contributions

VMM conceived and designed the study, developed the interview and survey questions, recruited participants, participated in the subject interviews, coded transcripts, and drafted the manuscript. MLM participated in the design of the study, participated in the subject interviews and helped to draft the manuscript. EC participated in the subject interviews and helped to draft the manuscript. All authors read and approved the final manuscript.

## Pre-publication history

The pre-publication history for this paper can be accessed here:



## References

[B1] Bass SA, Ferraro KF (2000). Gerontology education in transition: considering disciplinary and paradigmatic evolution. Gerontologist.

[B2] Alford CL, Miles T, Palmer R, Espino D (2001). An introduction to geriatrics for first year medical students. J Am Geriatr Soc.

[B3] Arnold L, Shue CK, Jones D (2002). Implementation of geriatric education into the first and second years of a baccalaureate-MD degree program. Acad Med.

[B4] Pan CX, Carmody S, Leipzig RM, Granieri E, Sullivan A, Block SD, Arnold RM (2005). There is hope for the future: national survey results reveal that geriatric medicine fellows are well-educated in end-of-life care. J Am Geriatr Soc.

[B5] Deary IJ, Smith R, Mitchell C, Mac Lennan WJ (1993). Geriatric medicine: does teaching alter medical students' attitudes to elderly people?. Med Educ.

[B6] Kaempfer D, Wellman NS, Himburg SP (2002). Dietetics students' low knowledge, attitudes, and work preferences toward older adults indicate need for improved education about aging. J Am Diet Assoc.

[B7] McKinlay A, Cowan S (2003). Student nurses' attitudes towards working with older patients. J Adv Nurs.

[B8] Wells Y, Foreman P, Gething L, Petralia W (2004). Nurses' attitudes toward aging and older adults-examining attitudes and practices among health services providers in Australia. J Gerontol Nurs.

[B9] Parse RR (2004). A human becoming teaching-learning model. Nurs Sci Q.

[B10] Letcher DC, Yancey NR (2004). Witnessing change with aspiring nurses: a human becoming teaching-learning process in nursing education. Nurs Sci Q.

[B11] (2002). American Psychological Association (APA): Resolution on ageism. http://www.apa.org/pi/aging/ageism.html.

[B12] Reuben DB, Fullerton JT, Tschann JM, Croughan-Minihane M (1995). Attitudes of beginning medical students toward older persons: a five-campus study. The University of California Academic Geriatric Resource Program Student Survey Research Group. J Am Geriatr Soc.

[B13] Cohen ES (2001). The complex nature of ageism: what is it? who does it? who perceives it?. Gerontologist.

[B14] Penson RT, Daniels KJ, Lynch TJ (2004). Too old care?. Oncologist.

[B15] Huberman AM, Miles MB, Denzin NK, Lincoln YS (1994). Data management and analysis methods. Handbook on qualitative research.

[B16] Diachun L, Hillier LM, Stolee P (2006). Interest in geriatric medicine in Canada: how can we secure a next generation of geriatricians?. J Am Geriatr Soc.

[B17] Fabiano JA, Waldrop DP, Nochajski TH, Davis EL, Goldberg LJ (2005). Understanding dental students' knowledge and perceptions of older people: toward a new model of geriatric dental education. J Dent Educ.

[B18] Diachun L, Dumbrell AC, Kerry Byrne K, Esbaugh J (2006). But does it stick? evaluating the durability of improved knowledge following an undergraduate experiential geriatrics learning session. J Am Geriatr Soc.

